# Supplementary material for: Macrophage signaling and function are regulated by distinct sterol biochemistries
Source: J Lipid Res. 2026 May 7;67(6):101054. doi: 10.1016/j.jlr.2026.101054 (PMC13254679; doi:10.1016/j.jlr.2026.101054)
Supplement: Supplemental data [file mmc1.pdf]

Supplemental Information

Macrophage signaling and function are regulated by distinct sterol biochemistries

Jazmine D. W. Yaeger, Jason G. Kerkvliet, Bijaya Pradhan, Amelia G. Lawver, Sonali Sengupta, Natalie W. Thiex, Kevin R. Francis

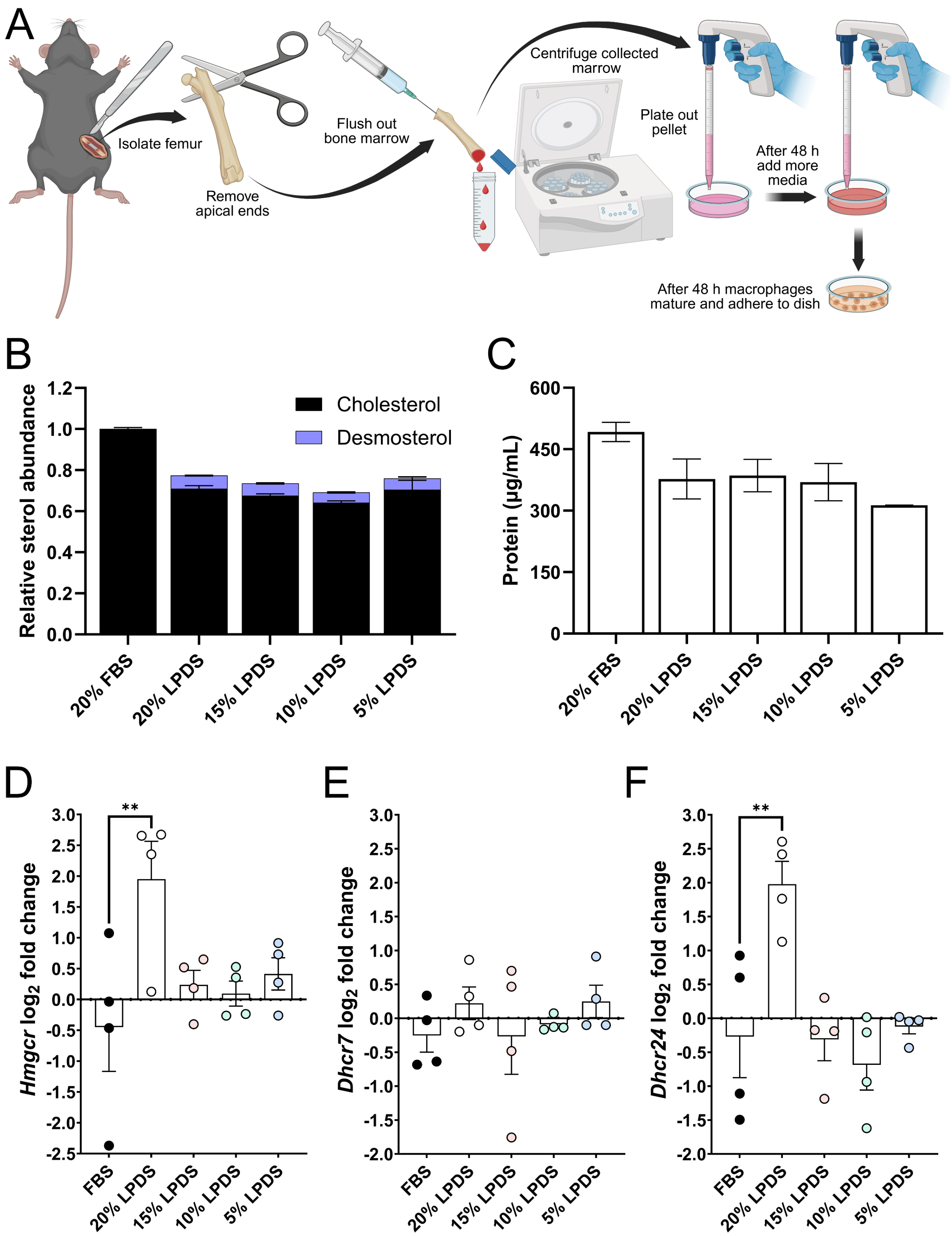
A
Isolate femur
Remove apical ends
Flush out bone marrow
Centrifuge collected marrow
Plate out pellet
After 48 h add more media
After 48 h macrophages mature and adhere to dish
B
Relative sterol abundance
Cholesterol
Desmosterol
20% FBS
20% LPDS
15% LPDS
10% LPDS
5% LPDS
C
Protein (µg/mL)
D
Hmgcr log2 fold change
FBS
E
Dhcr7 log2 fold change
F
Dhcr24 log2 fold change
**

Figure S1. Bone marrow-derived macrophages exhibit differential sterol profiles upon culture in lipoprotein deficient conditions. Related to Figure 1.

(A) Illustration summarizing the protocol for BMDM isolation from mouse femur.
(B) BMDMs cultured in LPDS for 48 h at varying concentrations exhibit reduced cellular cholesterol and desmosterol accumulation (mean ± SEM; n = 2 biological replicates from 2 independent experiments).
(C) Normalized protein content in BMDMs after incubation in various LPDS concentrations for 48 h (mean ± SEM; n = 2 biological replicates from 2 independent experiments).
(D) Quantified *Hmgcr* expression in BMDMs in FBS versus LPDS conditions for 48 h (mean ± SEM; n = 4 biological replicates from 2 independent experiments). One-way ANOVA ($F_{4,15} = 3.83$, $p \leq 0.0244$) with Dunnett's multiple comparisons test (**$p < 0.01$ compared to FBS).
(E) Quantified *Dhcr7* levels in FBS versus LPDS conditions for 48 h (mean ± SEM; n = 4 biological replicates from 2 independent experiments). One-way ANOVA ($F_{4,15} = 0.6399$, $p = 0.6421$).
(F) Quantified *Dhcr24* expression in FBS versus LPDS conditions for 48 h (mean ± SEM; n = 4 biological replicates from 2 independent experiments). One-way ANOVA ($F_{4,15} = 7.751$, $p \leq 0.0014$) with Dunnett's multiple comparisons test (**$p < 0.01$ compared to FBS).

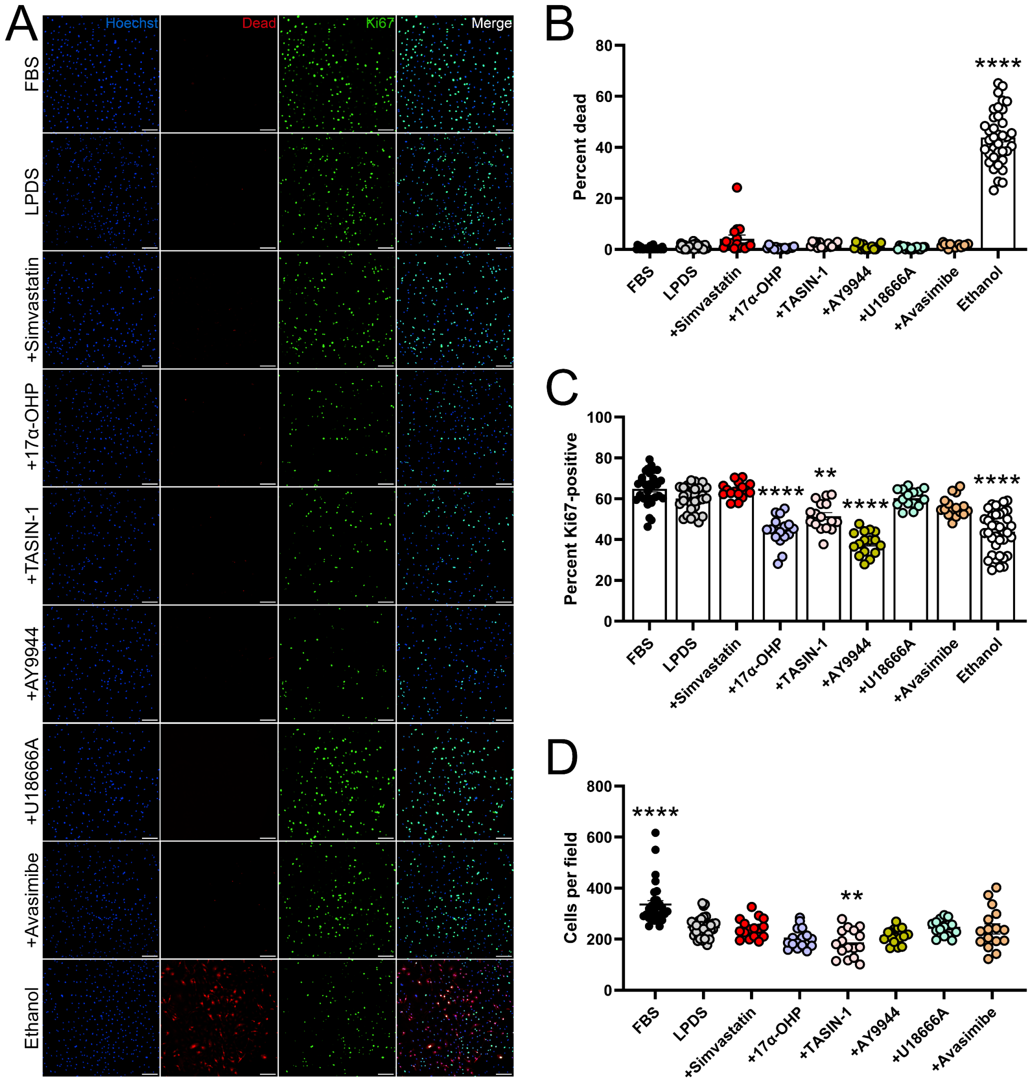

Figure S2. Impaired cholesterol biosynthesis inhibits macrophage proliferation. Related to Figure 1.

(A) Representative images of cell viability (red) and proliferative capacity (Ki67; green) in BMDMs. Hoechst counterstain is blue. Scale bar, 100 µm.

(B) Quantified cell death following inhibition of cholesterol biosynthesis for 48 h (mean ± SEM; n = 15-37 images taken from 3 independent experiments). One-way ANOVA ($F_{8,185} = 270.1$, $p < 0.0001$) with Dunnett's multiple comparisons test (****$p < 0.0001$ compared to LPDS).

(C) Quantified percent Ki67 positive cells following inhibition of cholesterol biosynthesis for 48 h (mean ± SEM; n = 15-37 images taken from 3 independent experiments). One-way ANOVA ($F_{8,185} = 40.21$, $p < 0.0001$) with Dunnett's multiple comparisons test (**$p < 0.01$; ****$p < 0.0001$ compared to LPDS).

(D) Quantified BMDMs per field when cultured with LPDS, FBS, or cholesterol biosynthesis inhibitors (mean ± SEM; n = 15-32 images taken from 3 independent experiments). One-way ANOVA ($F_{7,149} = 16.21$, $p < 0.0001$) with Dunnett's multiple comparisons test (**$p < 0.01$; ****$p < 0.0001$ compared to LPDS).

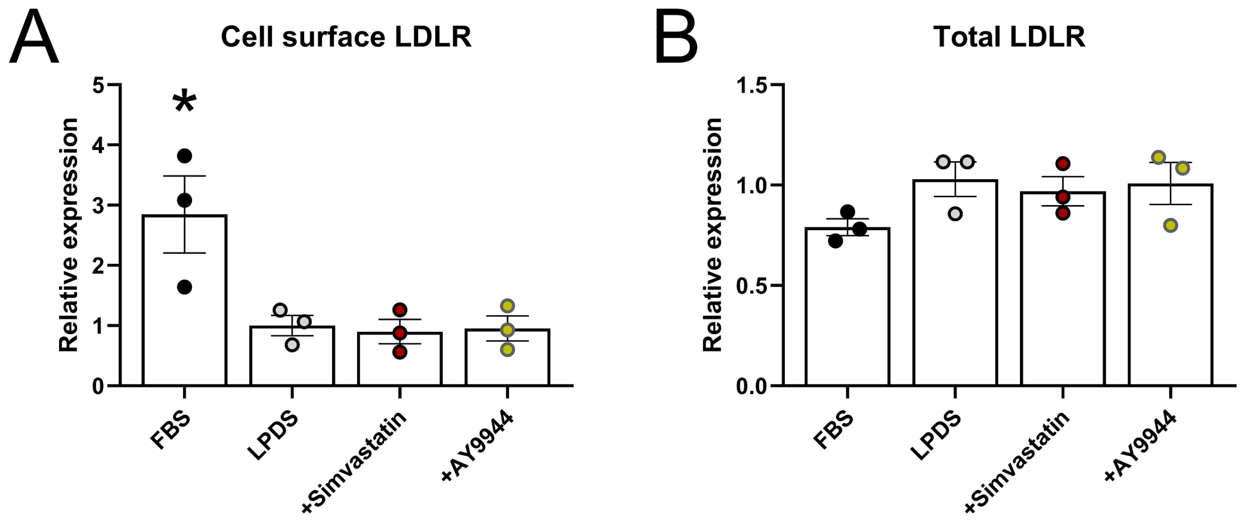

Figure S3. Inhibition of cholesterol biosynthesis in macrophages prevents surface expression of LDLR. Related to Figure 2.

(A) Cell surface expression of LDLR in cholesterol biosynthesis-inhibited BMDMs. Data shown as relative to LPDS (mean ± SEM; n = 3 biological replicates from 3 independent experiments). One-way ANOVA ($F_{3,8} = 6.896$, $p \leq 0.0131$) with Dunnett's multiple comparisons test (*$p \leq 0.0172$).

(B) Total expression of LDLR in cholesterol biosynthesis-inhibited BMDMs. Data shown as relative to LPDS (mean ± SEM; n = 3 biological replicates from 3 independent experiments).

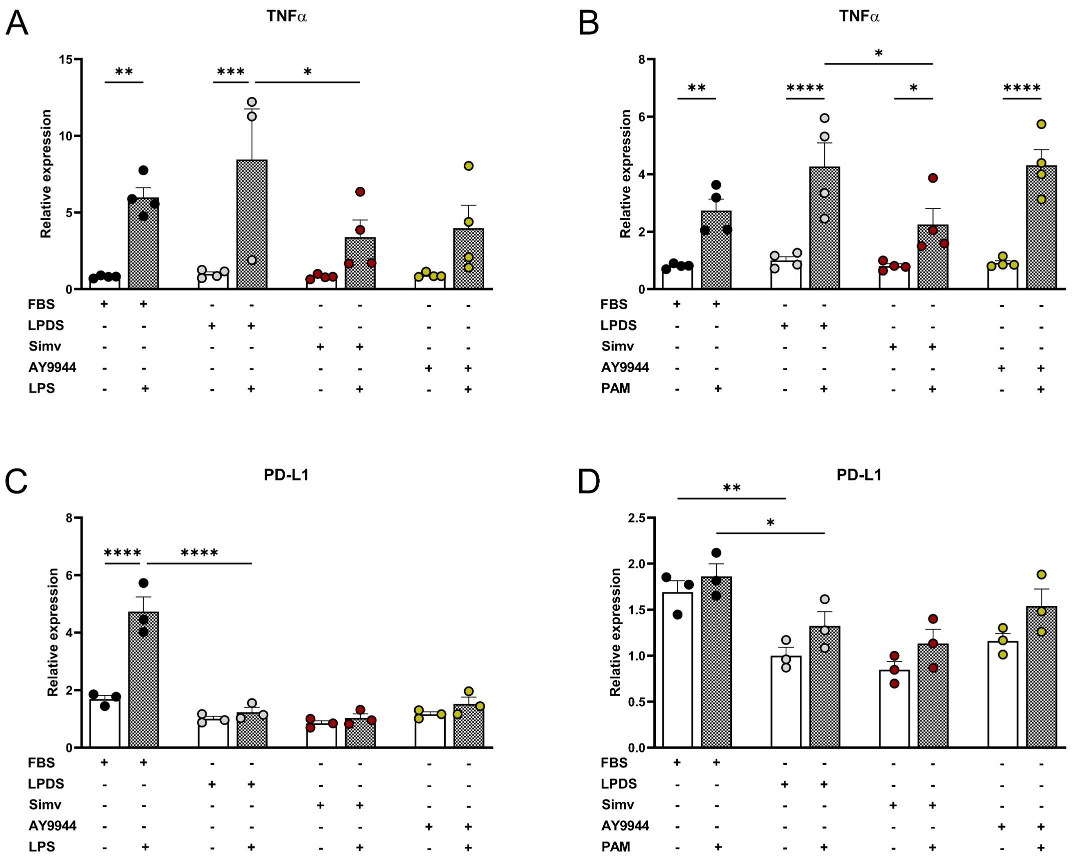

Figure S4. Disruption of macrophage cholesterol biosynthesis alters immune signaling. Related to Figure 2.

(A) TNFα expression in BMDMs with or without LPS activation. Data shown as relative to LPDS unstimulated conditions (mean ± SEM; n = 3-4 biological replicates from 4 independent experiments). Two-way ANOVA (stimulation effect: $F_{1,23} = 33.06$, $p < 0.0001$) with Tukey's multiple comparisons test (*$p \leq 0.05$, **$p \leq 0.01$, ***$p \leq 0.001$).

(B) TNFα expression in BMDMs with or without PAM activation. Data shown as relative to unstimulated LPDS conditions (mean ± SEM; n = 4 biological replicates from 4 independent experiments). Two-way ANOVA (stimulation effect: $F_{1,24} = 68.88$, $p < 0.0001$; treatment effect: $F_{3,24} = 3.555$, $p \leq 0.0293$) with Tukey's multiple comparisons test (*$p \leq 0.05$, **$p \leq 0.01$, ****$p < 0.0001$).

(C) PD-L1 expression in BMDMs with or without LPS activation. Data shown relative to unstimulated LPDS conditions (mean ± SEM; n = 3 biological replicates from 3 independent experiments). Two-way ANOVA (stimulation effect: $F_{1,16} = 36.46$, $p < 0.0001$; treatment effect: $F_{3,16} = 44.13$, $p < 0.0001$; interaction effect: $F_{3,16} = 19.35$, $p < 0.0001$) with Tukey's multiple comparisons test (****$p < 0.0001$).

(D) PD-L1 expression in BMDMs with or without PAM activation. Data shown as relative to LPDS unstimulated conditions (mean ± SEM; n = 3 biological replicates from 3 independent experiments). Two-way ANOVA (stimulation effect: $F_{1,16} = 9.856$, $p \leq 0.0063$; treatment effect: $F_{3,16} = 13.29$, $p < 0.0001$) with Tukey's multiple comparisons test (*$p \leq 0.05$, **$p \leq 0.01$).

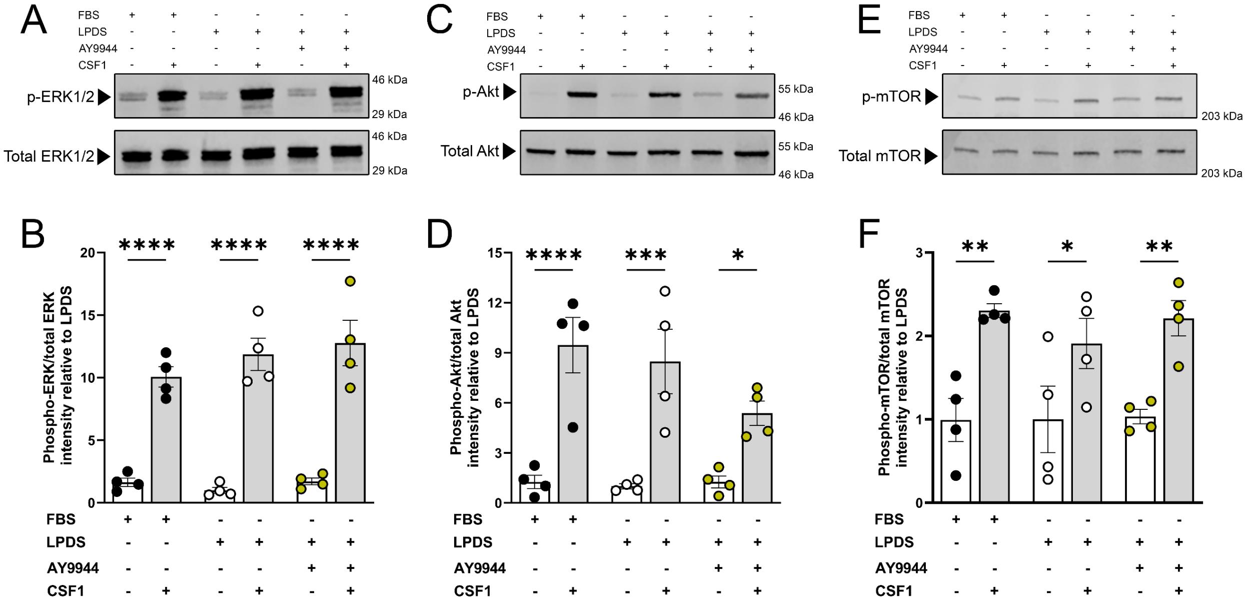

Figure S5. Impaired cholesterol biosynthesis does not significantly affect activation of signaling pathways critical to macrophage survival, proliferation, growth, and metabolism. Related to Figure 2.

(A) Representative immunoblot for ERK1/2 activation (phosphorylated and total ERK1/2).

(B) Ratio of phosphorylated ERK1/2 to total ERK1/2 after cholesterol depletion and CSF1 activation. Data shown relative to unstimulated LPDS conditions (mean ± SEM; n = 4 replicates from 4 independent experiments). Two-way ANOVA (CSF1 stimulation effect: $F_{1,18} = 156.0$, $p < 0.0001$) with Tukey's multiple comparisons test (****$p < 0.0001$).

(C) Representative immunoblot for Akt activation (phosphorylated and total Akt).

(D) Ratio of phosphorylated Akt to total Akt after cholesterol depletion and CSF1 activation. Data shown relative to unstimulated LPDS conditions (mean ± SEM; n = 4 replicates from 4 independent experiments). Two-way ANOVA (CSF1 stimulation effect: $F_{1,18} = 53.29$, $p < 0.0001$) with Tukey's multiple comparisons test (*$p \leq 0.05$; ***$p \leq 0.001$; ****$p < 0.0001$).

(E) Representative immunoblot for mTOR activation (phosphorylated and total mTOR).

(F) Ratio of phosphorylated mTOR to total mTOR after cholesterol depletion and CSF1 activation. Data shown relative to unstimulated LPDS conditions (mean ± SEM; n = 4 replicates from 4 independent experiments). Two-way ANOVA (CSF1 stimulation effect: $F_{1,18} = 30.7$, $p < 0.0001$) with Tukey's multiple comparisons test (*$p \leq 0.05$; **$p \leq 0.01$).

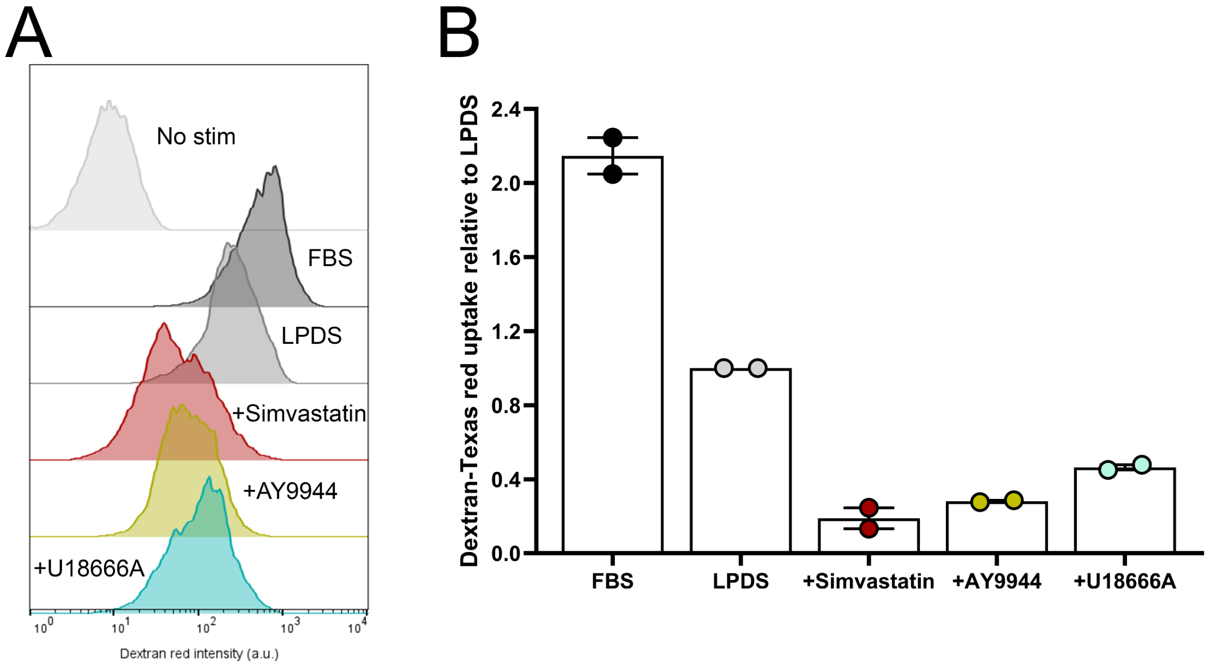

Figure S6. Clathrin-mediated endocytosis is attenuated in macrophages upon cholesterol homeostasis loss. Related to Figure 3.

(A) Representative histograms for Texas red dextran uptake in BMDMs in the presence or absence of cholesterol biosynthesis inhibition.

(B) Quantified Texas red dextran internalization after inhibition of cholesterol biosynthesis (mean ± SEM; n = 2 biological replicates from 2 independent experiments).

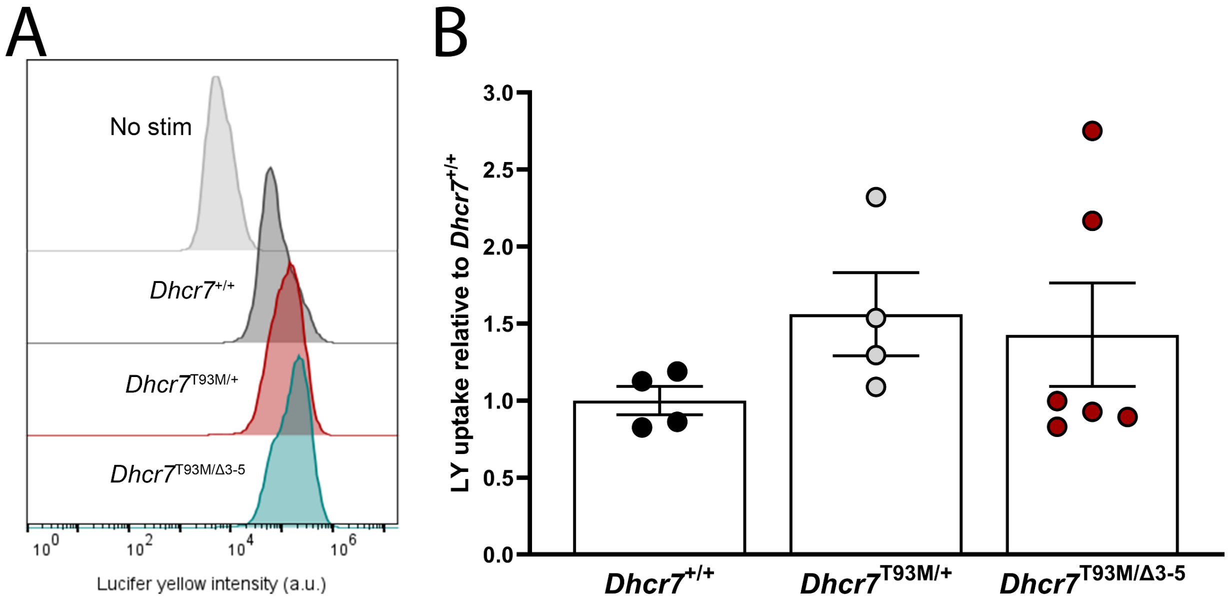

Figure S7 Macrophages from mouse models of Smith-Lemli-Opitz syndrome exhibit normal macropinocytic function in cholesterol rich conditions. Related to Figure 4.

(A) Representative histograms for LY uptake in *Dhcr7*$^{+/+}$, *Dhcr7*$^{T93M/+}$, and *Dhcr7*$^{T93M/\Delta3\text{-}5}$ BMDMs cultured in FBS conditions.

(B) Quantified LY uptake in *Dhcr7*$^{+/+}$, *Dhcr7*$^{T93M/+}$, and *Dhcr7*$^{T93M/\Delta3\text{-}5}$ BMDMs maintained in FBS conditions (mean ± SEM; n = 4-6 biological replicates from 4 independent experiment). One-way ANOVA ($F_{2,11} = 0.8875$, $p = 0.4392$).

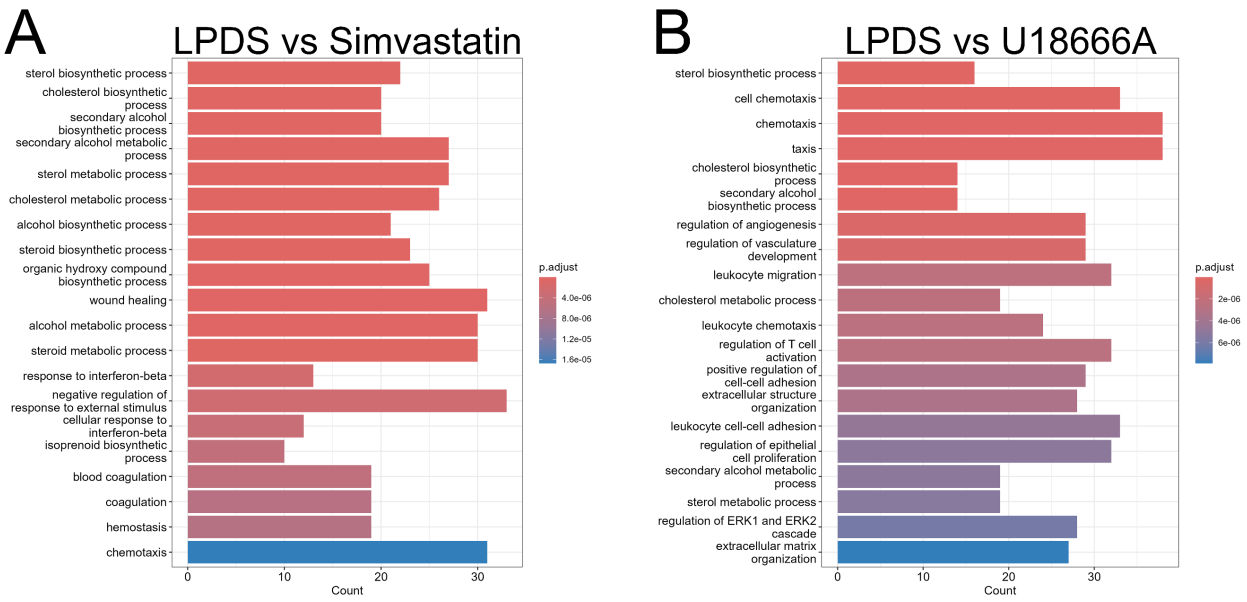

Figure S8. Pathway analyses of cholesterol biosynthesis impacts on macrophage signaling. Related to Figure 5.

(A) Pathway analysis of simvastatin treatment compared to LPDS conditions indicate altered sterol biosynthetic and immune response pathways (n = 4 biological replicates).

(B) Pathway analysis of U18666A treatment compared to LPDS culture revealed enhanced sterol biosynthetic and immune response after U18666A treatment (n = 4 biological replicates).

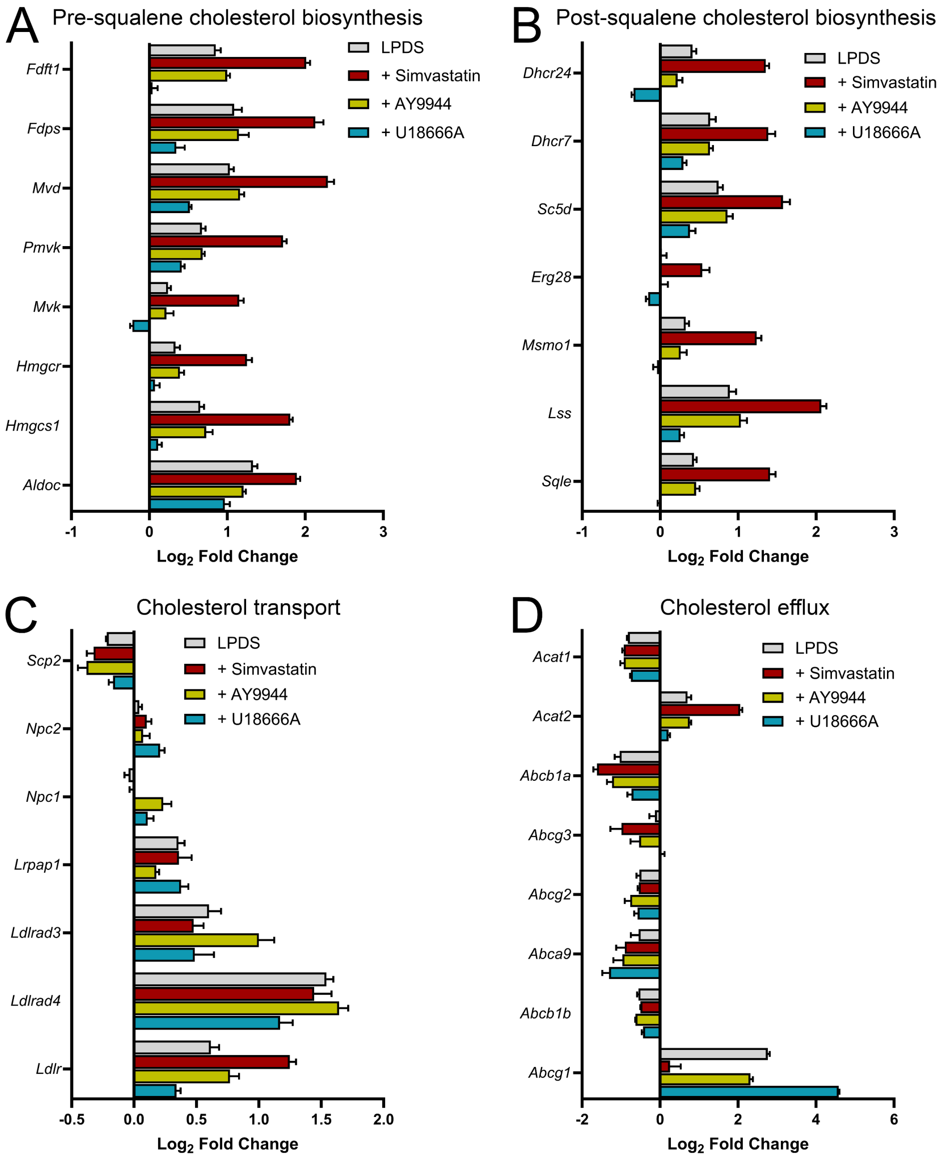

Figure S9. Impaired cholesterol biosynthesis in macrophages alters expression of sterol metabolism-associated transcripts. Related to Figure 5.

(A) Transcripts associated with activation of pre-squalene cholesterol biosynthesis are increased in treatments that reduce cholesterol biosynthesis (mean ± SEM; n = 4 biological replicates).

(B) Transcripts associated with post-squalene cholesterol biosynthesis are increased in conditions of impaired sterol metabolism (mean ± SEM; n = 4 biological replicates).

(C) Transcripts associated with regulation of cholesterol synthesis and transport are altered in response to cholesterol changes in macrophages (mean ± SEM; n = 4 biological replicates).

(D) Cholesterol deficient conditions induce transcriptional changes related to cholesterol efflux and lipid storage (mean ± SEM; n = 4 biological replicates).

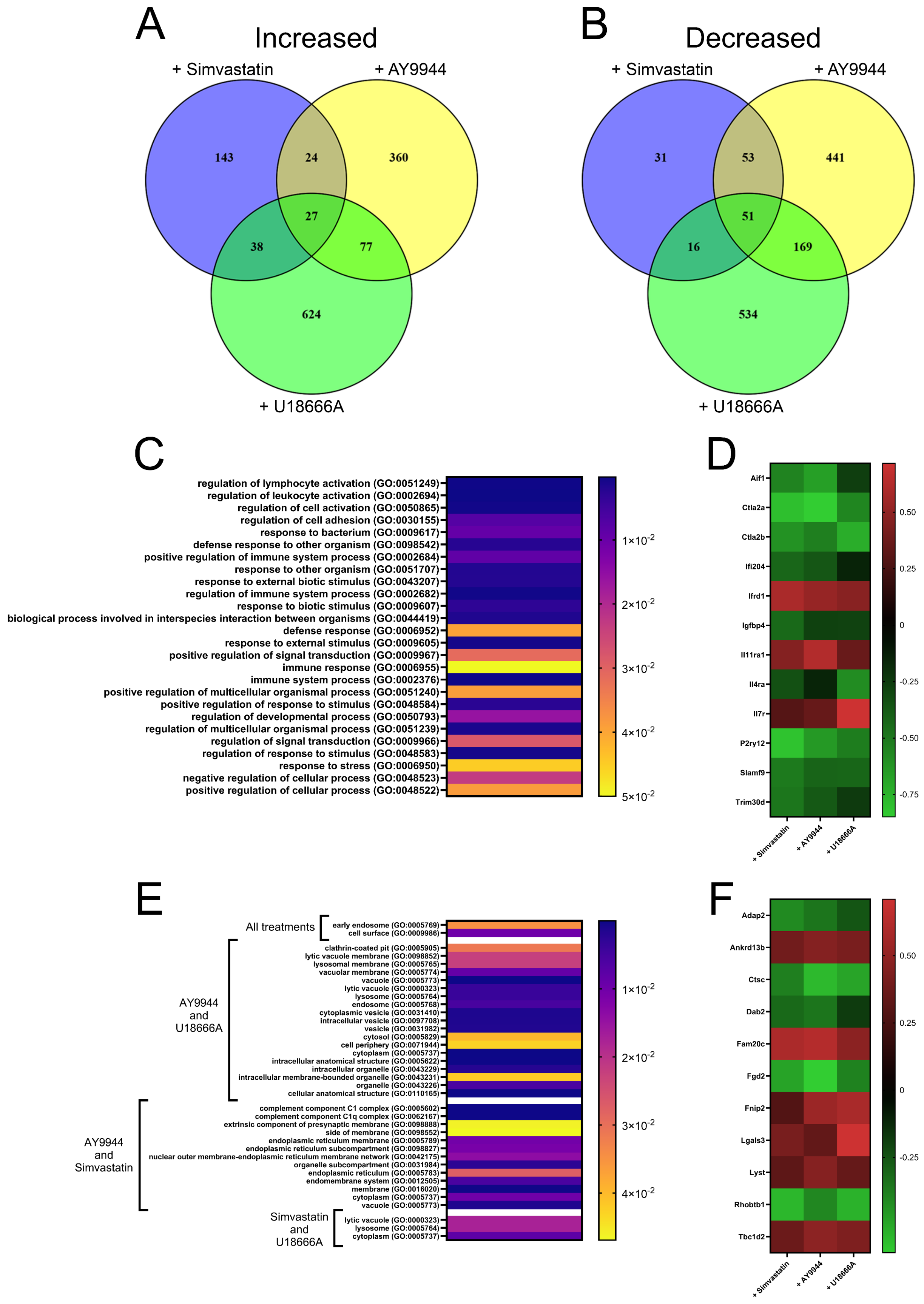

A
Increased
+ Simvastatin
+ AY9944
+ U18666A
143
24
360
27
38
77
624
B
Decreased
+ Simvastatin
+ AY9944
+ U18666A
31
53
441
51
16
169
534
C
regulation of lymphocyte activation (GO:0051249)
regulation of leukocyte activation (GO:0002694)
regulation of cell activation (GO:0050865)
regulation of cell adhesion (GO:0030155)
response to bacterium (GO:0009617)
defense response to other organism (GO:0098542)
positive regulation of immune system process (GO:0002684)
response to other organism (GO:0051707)
response to external biotic stimulus (GO:0043207)
regulation of immune system process (GO:0002682)
response to biotic stimulus (GO:0009607)
biological process involved in interspecies interaction between organisms (GO:0044419)
defense response (GO:0006952)
response to external stimulus (GO:0009605)
positive regulation of signal transduction (GO:0009967)
immune response (GO:0006955)
immune system process (GO:0002376)
positive regulation of multicellular organismal process (GO:0051240)
positive regulation of response to stimulus (GO:0048584)
regulation of developmental process (GO:0050793)
regulation of multicellular organismal process (GO:0051239)
regulation of signal transduction (GO:0009966)
regulation of response to stimulus (GO:0048583)
response to stress (GO:0006950)
negative regulation of cellular process (GO:0048523)
positive regulation of cellular process (GO:0048522)
1×10^{-2}
2×10^{-2}
3×10^{-2}
4×10^{-2}
5×10^{-2}
D
Aif1
Ctla2a
Ctla2b
Ifi204
Ifrd1
Igfbp4
Il11ra1
Il4ra
Il7r
P2ry12
Slamf9
Trim30d
+ Simvastatin
+ AY9944
+ U18666A
0.50
0.25
0
-0.25
-0.50
-0.75
E
All treatments
early endosome (GO:0005769)
cell surface (GO:0009986)
AY9944 and U18666A
clathrin-coated pit (GO:0005905)
lytic vacuole membrane (GO:0098852)
lysosomal membrane (GO:0005765)
vacuolar membrane (GO:0005774)
vacuole (GO:0005773)
lytic vacuole (GO:0000323)
lysosome (GO:0005764)
endosome (GO:0005768)
cytoplasmic vesicle (GO:0031410)
intracellular vesicle (GO:0097708)
vesicle (GO:0031982)
cytosol (GO:0005829)
cell periphery (GO:0071944)
cytoplasm (GO:0005737)
intracellular anatomical structure (GO:0005622)
intracellular organelle (GO:0043229)
intracellular membrane-bounded organelle (GO:0043231)
organelle (GO:0043226)
cellular anatomical structure (GO:0110165)
AY9944 and Simvastatin
complement component C1 complex (GO:0005602)
complement component C1q complex (GO:0062167)
extrinsic component of presynaptic membrane (GO:0098888)
side of membrane (GO:0098552)
endoplasmic reticulum membrane (GO:0005789)
endoplasmic reticulum subcompartment (GO:0098827)
nuclear outer membrane-endoplasmic reticulum membrane network (GO:0042175)
organelle subcompartment (GO:0031984)
endoplasmic reticulum (GO:0005783)
endomembrane system (GO:0012505)
membrane (GO:0016020)
cytoplasm (GO:0005737)
vacuole (GO:0005773)
Simvastatin and U18666A
lytic vacuole (GO:0000323)
lysosome (GO:0005764)
cytoplasm (GO:0005737)
1×10^{-2}
2×10^{-2}
3×10^{-2}
4×10^{-2}
F
Adap2
Ankrd13b
Ctsc
Dab2
Fam20c
Fgd2
Fnip2
Lgals3
Lyst
Rhobtb1
Tbc1d2
+ Simvastatin
+ AY9944
+ U18666A
0.50
0.25
0
-0.25

Figure S10. Gene Ontology pathway analyses reveal impacts of cholesterol biosynthesis inhibition on macrophage immune responsivity and vesicular trafficking processes. Related to Figure 5.

(A) Venn diagram illustrating transcripts significantly increased after cholesterol biosynthesis inhibition relative to LPDS conditions (n = 4 biological replicates per condition; $\log_2$ fold change $\geq \pm 0.5$, $p < 0.05$).
(B) Venn diagram showing transcripts significantly decreased after disrupted cholesterol biosynthesis relative to LPDS conditions (n = 4 biological replicates per condition; $\log_2$ fold change $\geq \pm 0.5$, $p < 0.05$).
(C) GO pathway analyses for biological processes of shared transcripts suggest immune-related dysfunction in macrophages treated with cholesterol biosynthesis inhibitors relative to LPDS conditions. (n = 4 biological replicates per condition).
(D) Heat map showing changes in selected transcripts related to immune response that are differentially expressed in simvastatin, AY9944, and U18666A treatments relative to LPDS control conditions (mean ± SEM; n = 4 biological replicates per condition).
(E) GO pathway analyses for cellular components of shared transcripts show disruption of vesicular trafficking pathways in macrophages treated with cholesterol biosynthesis inhibitors relative to LPDS conditions. (n = 4 biological replicates per condition).
(F) Heat map showing selected transcripts associated with intracellular vesicular trafficking that are differentially expressed in simvastatin, AY9944, and U18666A treatments relative to LPDS conditions (mean ± SEM; n = 4 biological replicates per condition).

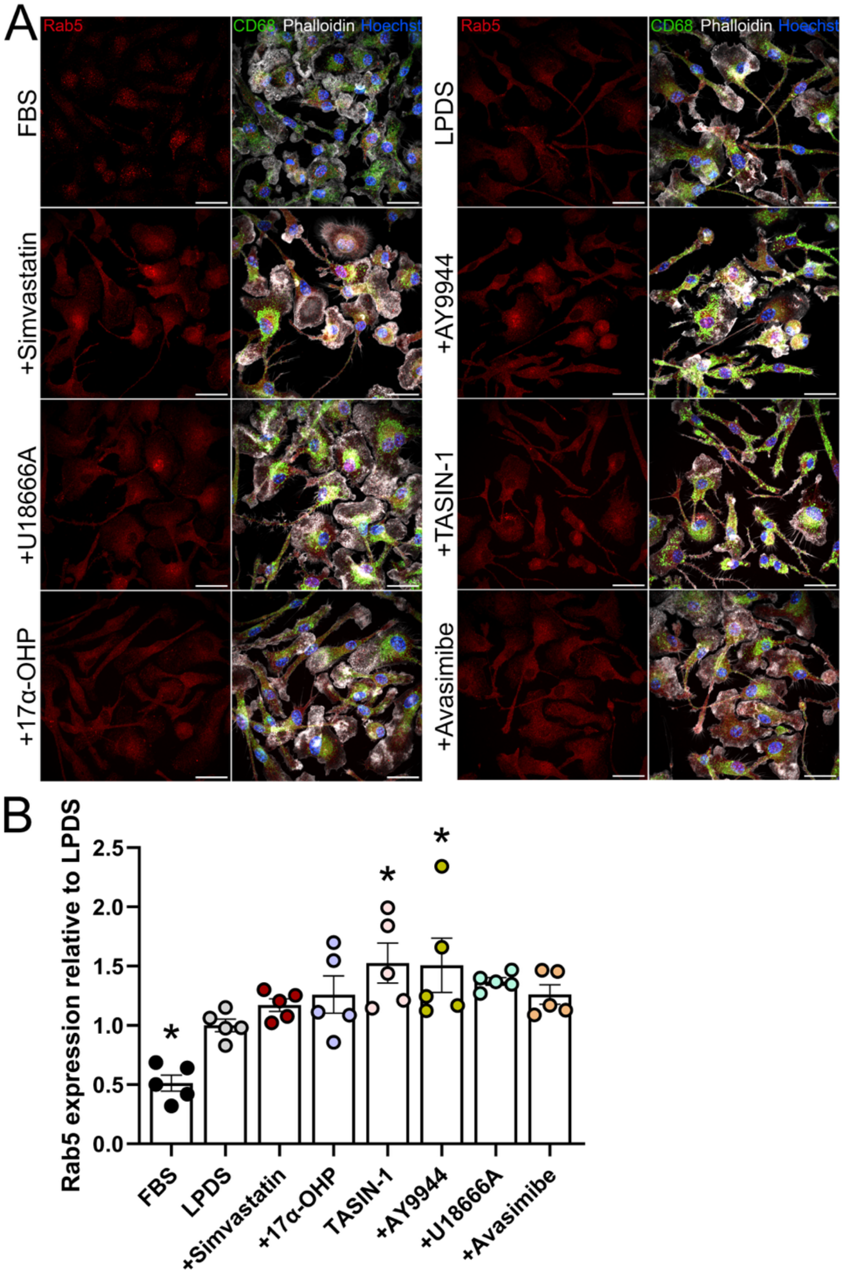

Figure S11. Dysregulation of cholesterol biosynthesis impacts early endosomal pathways. Related to Figure 6.

(A) Representative images of Rab5 expression with CD68, phalloidin-647, and Hoechst counterstain in BMDMs. Scale bar, 25 µm.

(B) Analysis of Rab5 expression after cholesterol synthesis antagonism relative to control conditions (mean ± SEM; n = 5 images taken from 1 independent experiment). One-way ANOVA ($F_{7,32}$ = 6.921, $p < 0.0001$) with Dunnett's multiple comparisons test (*$p < 0.05$ compared to LPDS conditions).

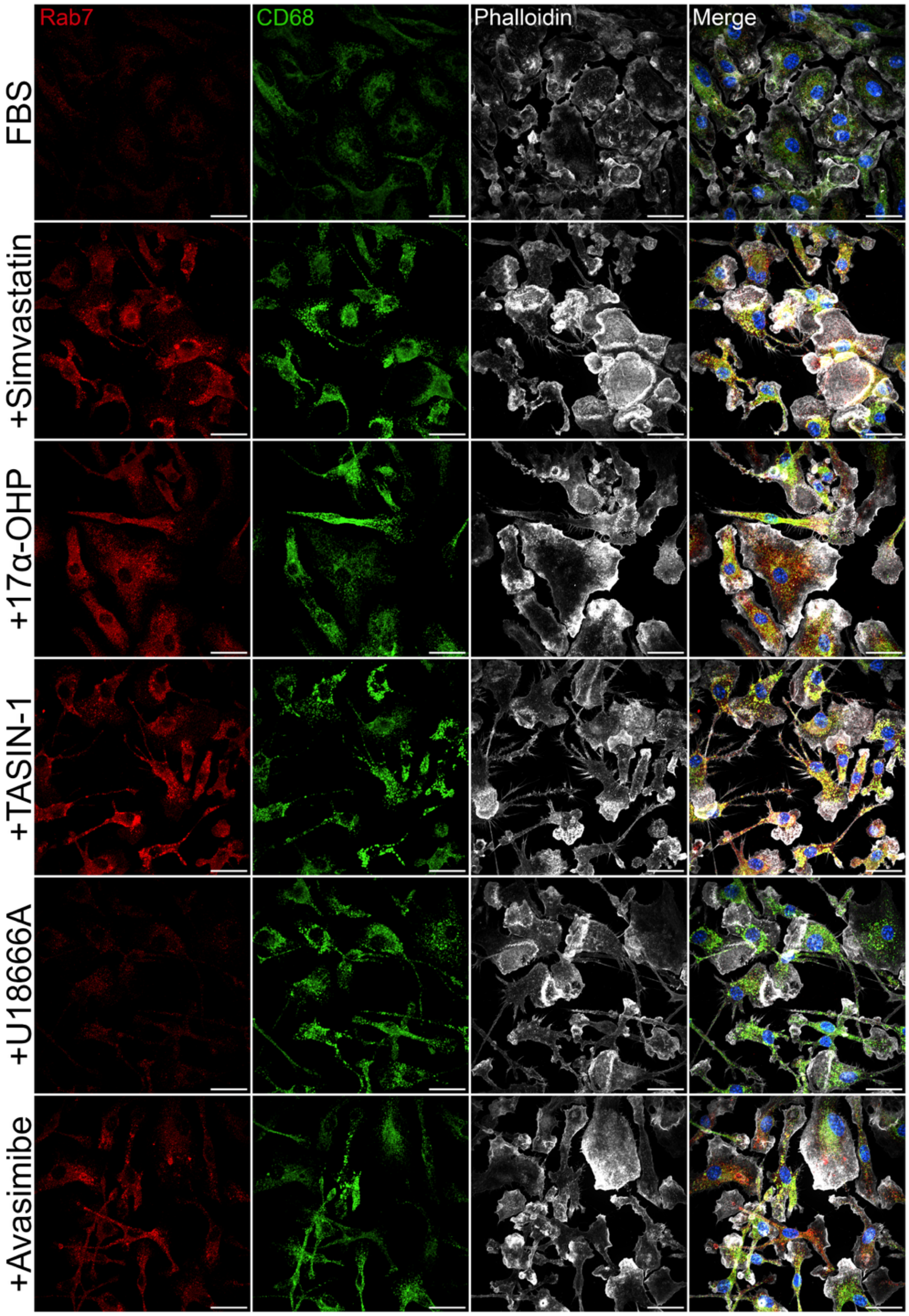

Figure S12. Disruption of cholesterol metabolism impacts intracellular vesicle trafficking in macrophages. Related to Figure 6.

Representative images of markers of late endosomes/lysosomes (Rab7, CD68) and F-actin filaments (phalloidin) in BMDMs with or without cholesterol biosynthesis inhibition. Hoechst counterstain is blue. Scale bar, 25 μm.

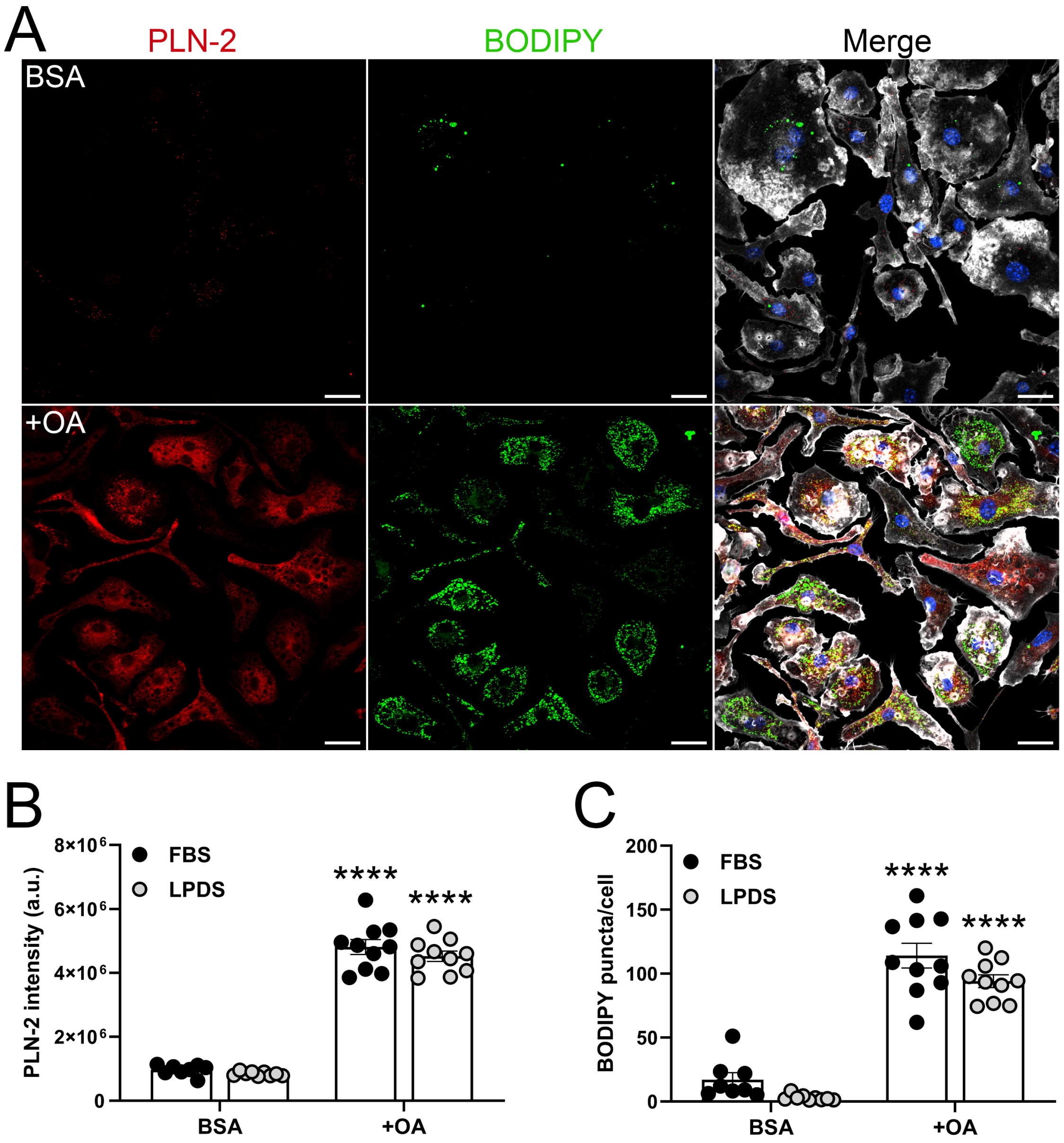

Figure S13. Lipoprotein deficient culture does not prevent macrophage lipid storage. Related to Figure 6.

(A) Representative images of PLN-2 and BODIPY 505/515 in LPDS cultured BMDMs after BSA or oleic acid (OA) treatment. Hoechst counterstain is blue. Scale bar, 25 µm.

(B) Quantified PLN-2 expression normalized by cell count after OA supplementation compared to BSA in FBS and LPDS conditions (mean ± SEM; n = 8-10 images taken from 2 independent experiments). Two-way ANOVA (Media effect: $F_{1,33} = 571.6$, $p < 0.0001$) with Tukey's multiple comparisons test (****$p < 0.0001$ compared to BSA).

(C) Neutral lipid puncta labeled with BODIPY 505/515 are increased after OA treatment compared to BSA control conditions in both FBS and LPDS conditions (mean ± SEM; n = 8-10 images taken from 2 independent experiments). Two-way ANOVA (Media effect: $F_{1,33} = 217.2$, $p < 0.0001$; OA effect: $F_{1,33} = 7.212$, $p < 0.0112$) with Tukey's multiple comparisons test (****$p < 0.0001$ compared to BSA).

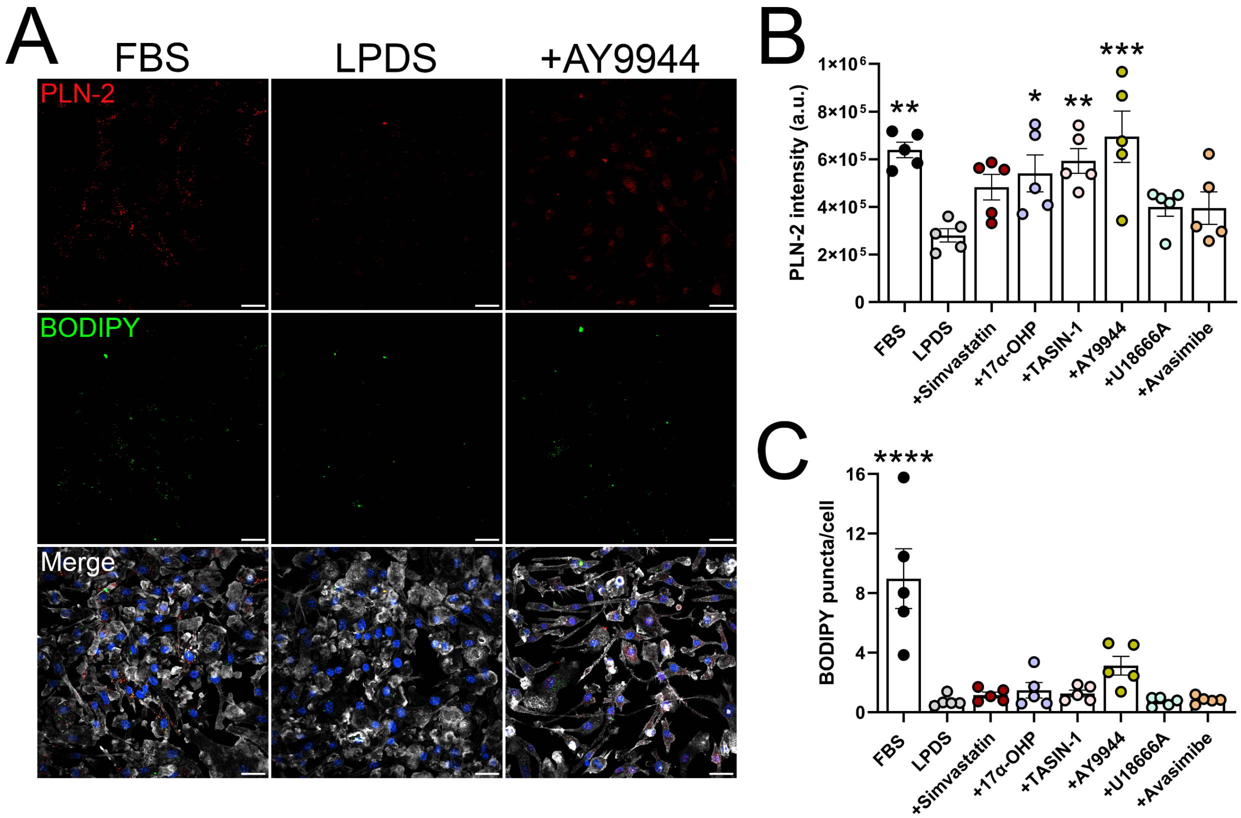

Figure S14. Disrupted cholesterol biosynthesis does not promote lipid droplet formation in macrophages. Related to Figure 6.

(A) Representative images of PLN-2 and BODIPY 505/515 in FBS, LPDS, or AY9944 cultured BMDMs. Scale bar, 25 µm.

(B) Quantified PLN-2 expression normalized by cell count in BMDMs with or without inhibition of cholesterol metabolism (mean ± SEM; n = 5 images taken from 1 independent experiment). One-way ANOVA ($F_{7,32} = 5.006$, $p \leq 0.0007$) with Dunnett's multiple comparisons test (*$p \leq 0.05$; **$p \leq 0.01$; ***$p \leq 0.001$ compared to LPDS).

(C) Quantified BODIPY 505/515 puncta in BMDMs with or without inhibition of cholesterol metabolism (mean ± SEM; n = 5 images taken from 1 independent experiment). One-way ANOVA ($F_{7,32} = 13.11$, $p < 0.0001$) with Dunnett's multiple comparisons test (****$p < 0.001$ compared to LPDS).

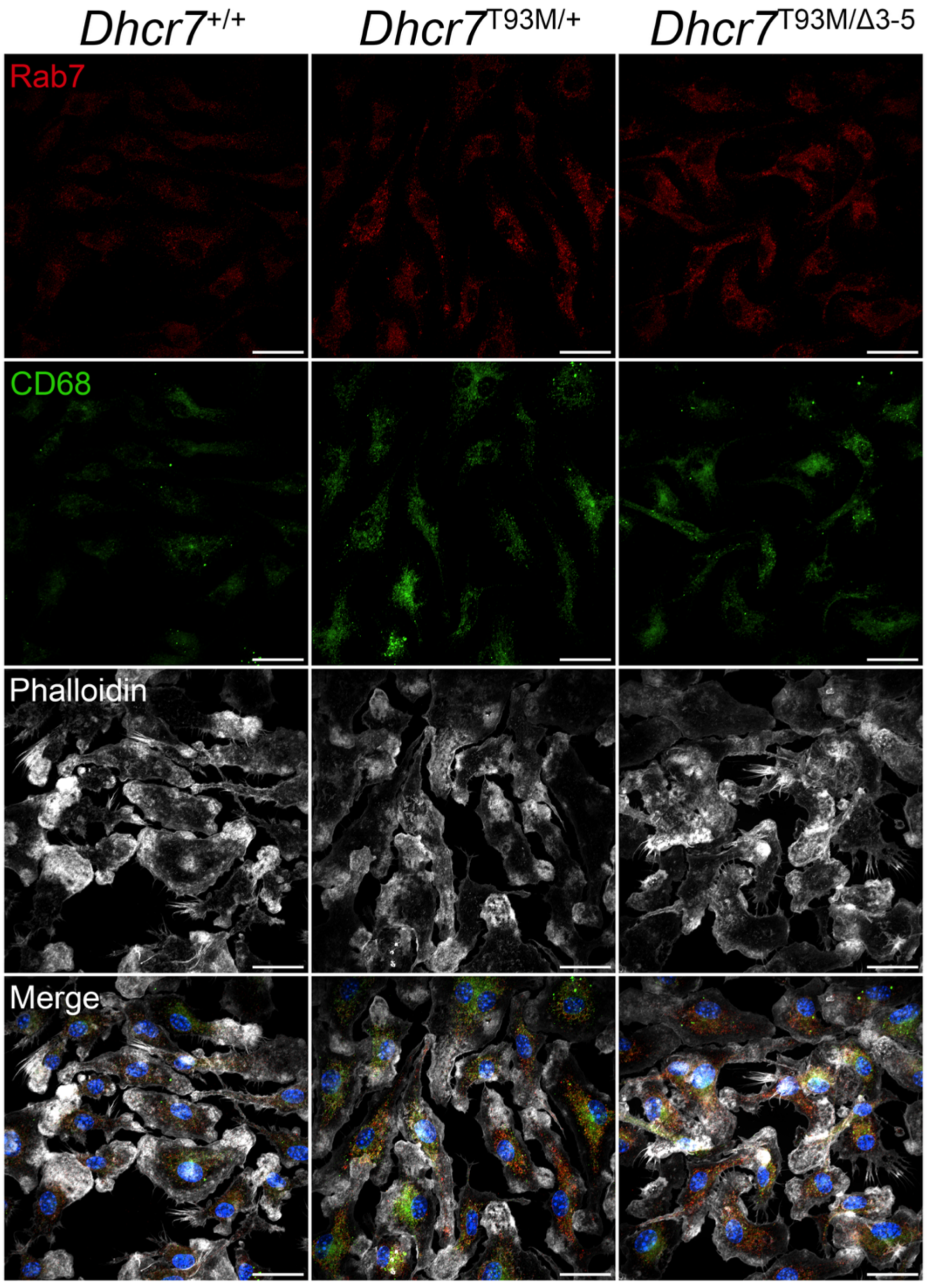

Figure S15. Macrophages derived from mouse models of Smith-Lemli-Opitz syndrome display disrupted trafficking of intracellular vesicles. Related to Figure 7.

Representative images of markers of late endosomes/lysosomes (Rab7, CD68) in FBS-cultured BMDMs derived from wild-type (*Dhcr7*$^{+/+}$) and mutant (*Dhcr7*$^{T93M/+}$, *Dhcr7*$^{T93M/\Delta 3\text{-}5}$) mice. Scale bar, 25 μm.

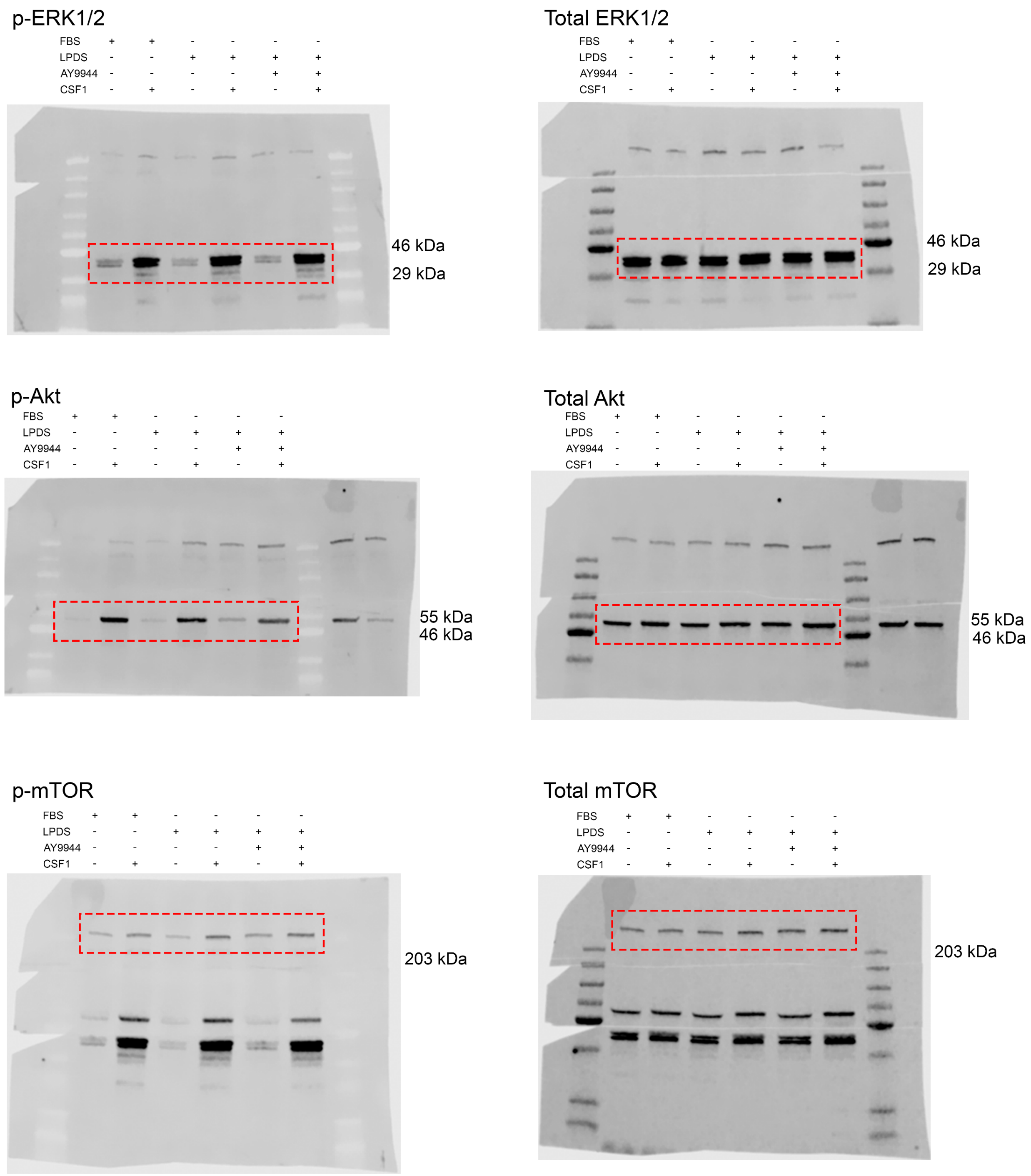

Figure S16. Unedited and uncropped western blots displayed in Figure S5.
